# The Role of Mitochondrial Function in Peripheral Arterial Disease: Insights from Translational Studies

**DOI:** 10.3390/ijms22168478

**Published:** 2021-08-06

**Authors:** Alexandra Gratl, Sabine Wipper, Jan Paul Frese, Ben Raude, Andreas Greiner, Dominik Pesta

**Affiliations:** 1 Department of Vascular Surgery, Medical University of Innsbruck, A-6020 Innsbruck, Austria; sabine.wipper@i-med.ac.at; 2 Department of Vascular Surgery, Charité—University Hospital of Berlin, D-12203 Berlin, Germany; jan-paul-bernhard.frese@charite.de (J.P.F.); ben-heinrich.raude@charite.de (B.R.); andreas.greiner@charite.de (A.G.); 3 German Aerospace Center (DLR), Institute of Aerospace Medicine, D-51147 Cologne, Germany; 4 Center for Endocrinology, Diabetes and Preventive Medicine (CEDP), University Hospital Cologne, D-50931 Cologne, Germany; 5 Cologne Excellence Cluster on Cellular Stress Responses in Aging-Associated Diseases (CECAD), D-50931 Cologne, Germany; 6 Institute for Clinical Diabetology, German Diabetes Center, Leibniz Center for Diabetes Research at Heinrich-Heine University Düsseldorf, D-40225 Düsseldorf, Germany; 7 German Center of Diabetes Research (DZD e.V.), D-85764 Neuherberg, Germany

**Keywords:** mitochondrial function, peripheral arterial disease, physical activity, mitochondrial recovery, revascularization

## Abstract

Recent evidence demonstrates an involvement of impaired mitochondrial function in peripheral arterial disease (PAD) development. Specific impairments have been assessed by different methodological in-vivo (near-infrared spectroscopy, ^31^P magnetic resonance spectroscopy), as well as in-vitro approaches (Western blotting of mitochondrial proteins and enzymes, assays of mitochondrial function and content). While effects differ with regard to disease severity, chronic malperfusion impacts subcellular energy homeostasis, and repeating cycles of ischemia and reperfusion contribute to PAD disease progression by increasing mitochondrial reactive oxygen species production and impairing mitochondrial function. With the leading clinical symptom of decreased walking capacity due to intermittent claudication, PAD patients suffer from a subsequent reduction of quality of life. Different treatment modalities, such as physical activity and revascularization procedures, can aid mitochondrial recovery. While the relevance of these modalities for mitochondrial functional recovery is still a matter of debate, recent research indicates the importance of revascularization procedures, with increased physical activity levels being a subordinate contributor, at least during mild stages of PAD. With an additional focus on the role of revascularization procedures on mitochondria and the identification of suitable mitochondrial markers in PAD, this review aims to critically evaluate the relevance of mitochondrial function in PAD development and progression.

## 1. Introduction

Peripheral arterial disease (PAD) is one of the various manifestations of arteriosclerotic diseases and affects more than 200 million people worldwide [1]. After the age of 65 years, the incidence increases exponentially, with men being affected more frequently than women [1]. With more than 450 million affected individuals to date, diabetes mellitus is one of the largest global public health concerns. Concerningly, diabetes mellitus remains a risk factor for all forms of cardiovascular diseases and, hence, also a strong risk factor for PAD [2].

Arteriosclerotic lesions within the arterial tree of the lower extremities initially present with clinical asymptomatic stenosis of lower extremity arteries and can progress to the most severe form of critical limb ischemia, which may in certain cases require limb amputation. Incidences of major amputations vary from 120 to 500 cases per million per year [3], and mortality directly related to PAD has constantly increased over the last few years [4]. Mild forms of PAD result in the typical clinical manifestation of intermittent claudication. As a result of flow-limiting arteriosclerotic lesions, blood supply to affected muscle regions is restricted, resulting in a walking-induced pain with subsequent limitation of walking performance. Depending on the maximal walking distance, the clinical stage of PAD is classified differently. If intermittent claudication is lifestyle limiting, corresponding to stage IIB PAD using the widely used Fontaine classifications system [5], treatment options, such as exercise training to increase collateral blood flow, as well as interventional revascularization of underlying lesions, are available. The indication for interventional treatment in these mild forms of PAD is optional, as affected limbs are not threatened in viability [6]. The goal of treatment of intermittent claudication is to reestablish functionality of affected limbs and to reestablish the patients’ quality of life. The direct correlation of an improvement of functional status and hemodynamic parameters after revascularization procedures was refuted by several authors in the past [7,8], leading to the assumption of chronic, irreversible alterations within muscle, even after restoration of blood supply. More severe stages of PAD include ischemic pain of affected limbs (stage III) at rest, as well as trophic lesions as a result of chronic malperfusion (stage IV) [5]. Invasive treatment with the aforementioned revascularization procedures is inevitable in these severe forms of PAD, in order to avoid further tissue damage.

Subsequent to understanding the pathophysiology of the stenosis with ensuing insufficient oxygen and nutrient supply to affected muscle regions, it was of great interest to identify underlying disease mechanisms and subcellular changes. With the onset of characteristic myopathies associated with PAD, mitochondria have been identified as crucial mediators in this process [9,10,11].

Mitochondria are responsible for aerobic energy production in all eukaryotic cells. As electrons from nicotinamide adenine dinucleotide (NADH) and flavin adenine dinucleotide (FADH_2_) pass through a sequence of protein complexes anchored to the inner mitochondrial membrane, an electrochemical gradient is established and harnessed to produce adenosine triphosphate (ATP) by the enzyme ATP synthase in the process of oxidative phosphorylation. Besides this key function, mitochondria also play a role in intracellular communication and apoptosis pathways, or reactive oxygen species (ROS) production [10]. With their distinct subcellular localization and their close proximity to other mitochondria [12], communication within a complex network and remodeling (“mitochondrial dynamics”) is possible [13]. Disruption of this complex network and subsequent impairments of mitochondrial function are associated with development and progression of PAD [9].

This review aims to summarize current knowledge on mitochondrial alterations in PAD with a specific focus on the effect of different treatment modalities and their impact on mitochondrial recovery.

## 2. Do Mitochondria Play a Role in the Pathogenesis of Ischemic Myopathy in PAD?

Morphological changes in muscle affected by chronic malperfusion are well known and pertain histomorphological modifications at the level of the myofibrils [14]. The severity of changes was associated with the severity of the disease. For example, Hedberg et al. described significant histomorphological differences in the gastrocnemius muscle after conservative therapy (exercise training) as compared to interventional revascularization procedures due to severity of disease [15]. Using electron microscopy techniques to further investigate subcellular changes of affected muscle regions, mitochondrial morphological alterations have been found to be associated with the pathophysiology of PAD [10].

Evidence for mitochondrial dysfunction in PAD stems from studies using in-vivo, as well as in-vitro, methods. Using ^31^P-magnetic resonance spectroscopy (MRS), real-time measurement of ATP, adenosine diphosphate (ADP), and phosphocreatine (PCr) concentrations were performed in patients with PAD and compared to healthy people. ATP supply is buffered by PCr breakdown, which is depleted during muscular contractions as it transfers its phosphate group to ADP to form ATP. The rephosphorylation of Cr takes place at the mitochondrion, using ATP derived from oxidative metabolism. Therefore, PCr recovery rates can serve as a surrogate measure of mitochondrial function [16]. Prolonged recovery rates in patients suffering from PAD compared to contralateral asymptomatic legs, as well as to those from healthy control persons, indicate impaired mitochondrial function in these individuals [17,18,19,20].

Using in-vitro respirometry, the group of Pipinos described an overall impairment of mitochondrial function in patients suffering from severe PAD [21]. This study, however, only involved a relatively small number of patients, and the authors did not assess mitochondrial content, which does not allow for differentiation of qualitative and quantitative changes. More in-depth analysis involving additional measurements of specific complexes of the respiratory chain, as well as to mitochondrial content and evaluation of oxidative stress biomarkers and antioxidant enzymes, revealed alterations of complexes I, III, and IV [22]. In addition, biomarkers for oxidative stress (protein carbonyl, lipid hydroperoxide, and 4-hydroxy-2-nonenal (HNE) levels) were significantly increased, and antioxidative capacity derived from mitochondrial superoxide dismutase activity was decreased in muscle samples from these patients [22]. Mitochondria are an important source of ROS, and alterations of mitochondrial function can, therefore, contribute to oxidative stress [23]. With complexes I and III being the main sites of mitochondrial ROS production [24,25], mitochondrial alterations can result in a vicious cycle of respiratory chain complex dysfunction. Taken together, limited blood supply as a result of arterial occlusion can result in dysfunction of the complexes of the respiratory chain within the myocyte with subsequent increase in ROS production and decreased antioxidant defense mechanisms, resulting in oxidative stress, which can lead to further impairments of the respiratory system [22].

**Figure 1:**
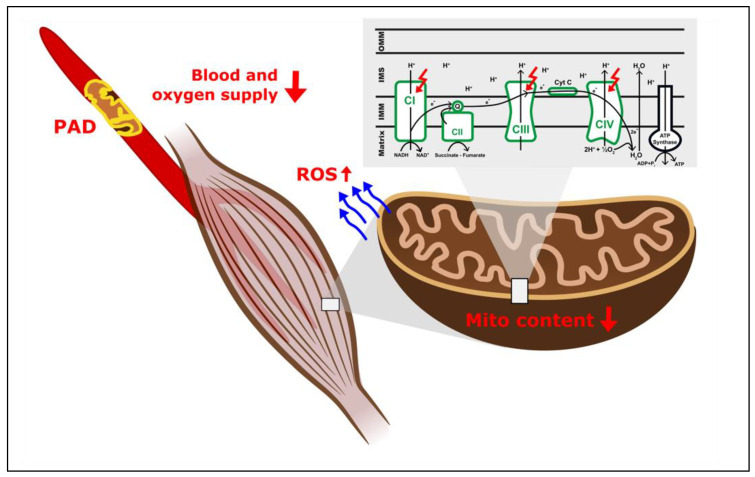
Alterations of mitochondrial function as a result of decreased blood and oxygen supply in PAD. ADP—adenosine diphosphate; ATP—adenosine triphosphate; CI—complex I; CII—complex II; CIII—complex III; CIV—complex IV; Cyt C—cytochrome C; e^-^—electron; H^+^—proton; H_2_O—water; IMM—inner mitochondrial membrane; IMS—intermembrane space; O_2_—oxygen; NADH/NAD^+^—nicotinamide adenine dinucleotide; OMM—outer mitochondrial membrane; PAD—peripheral arterial disease; P_i_—inorganic phosphate; Q—ubiquinone; ROS—reactive oxygen species.

Table 1 provides an overview of clinical studies investigating mitochondrial function in the context of PAD. The aforementioned alterations in mitochondria, following a decrease of blood and oxygen supply due to arteriosclerotic lesions in PAD are shown in Figure 1.

## 3. Effects of Ischemia Reperfusion Injury on Muscle Mitochondria

Besides the impact of chronic malperfusion on the cellular environment, ischemia reperfusion injury (IRI) is a reoccurring condition in PAD. During acute ischemia, removal of the obstruction, which is most commonly a blood clot within the arterial tree, is mandatory to prevent irreversible ischemic damage of affected tissue. Depending on the duration of ischemia and the type of affected tissue, reperfusion leads to different stages of cellular damage [26]. Reperfusion following ischemia is present in chronically affected muscle regions. Physical activity in patients suffering from intermittent claudication is limited due to walking-induced pain as a result of ischemia. Pain caused by ischemic conditions of affected muscle following physical activity tapers off once the activity ceases. These cycles of ischemia and reperfusion are repeated chronically and can harm the cellular environment of the affected myocytes.

Besides the recommendation to perform exercise training, the treatment of PAD involves interventional revascularization of underlying arteriosclerotic lesions. Flow-limiting stenosis or occlusions are treated by open surgical (endarteriectomy, bypass grafting) or endovascular procedures (balloon angioplasty with or without stenting) [6]. Restoration of blood supply results in reperfusion of chronic ischemic muscle regions.

Reestablishing perfusion of affected regions after acute ischemia can prevent irreversible tissue damage. Effects of IRI on muscle mitochondria are mainly based on findings from acute ischemic conditions. Effects of chronic ischemia-reperfusion cycles remain understudied and should be considered in future studies. Depending on tissue type and reperfusion time of ischemic tissue, different stages of cellular damage have been described [27,28,29]. Upon reperfusion, mitochondrial ROS production increases, resulting in oxidative damage to mitochondria and the surrounding cellular environment and disruption of ATP production [26]. Subsequently, intracellular acidosis as a result of ischemia is corrected by an increase of calcium release in response to reperfusion. This causes an opening of mitochondrial permeability transition pores and a sudden change in mitochondrial membrane permeability, resulting in a loss of membrane potential and release of pro-apoptotic factors and cell death [9,27,30,31,32]. Ischemia-reperfusion events impair mitochondrial and cellular integrity by increasing free radical production in combination with dysfunction of the complexes of the respiratory system, which results in lipid peroxidation, protein oxidation, and mitochondrial DNA damage [9].

## 4. Methods to Assess Mitochondrial Function in PAD

In order to assess mitochondrial function, various invasive and non-invasive technologies are available and used complementarily to one another, depending on the research question. Technical advancements allow for in-vivo analysis of metabolic processes or minimally invasive investigations of the subcellular milieu.

### 4.1. Near-Infrared Spectroscopy (NIRS)

Near-infrared spectroscopy (NIRS) enables the in-vivo visualization of the mismatch between oxygen demand and oxygen supply, especially during exercise, and distally of flow-limiting arteriosclerotic lesions [32]. NIRS uses near-infrared light with different wavelengths (700–900 nm) to transmit photons to the muscle of interest non-invasively as the probes are positioned on the skin of the respective region [33]. Adsorption rates of oxygenated and deoxygenated heme-containing molecules are dynamically measured. Using NIRS, oxygen demand and supply can be evaluated in healthy and diseased populations [34].

### 4.2. 31-Phosphorus Magnetic Resonance Spectroscopy (^31^P MRS)

^31^P is the only stable isotope of phosphorus and is part of oxidative phosphorylation as a component of high-energy phosphates. Within a magnetic field, it is excited with a specific frequency of 25.8 MHz and can, thus, be detected by MRS. ^31^P MRS is a non-invasive technology to study the high-energy phosphate metabolism, such as depletion and regeneration of PCr, in the muscle [34]. The recovery rates of PCr and ADP determined by ^31^P MRS serve as a surrogate marker for mitochondrial capacity. In patients with myopathy, recovery rates of these metabolites are slower compared to healthy individuals, indicating impairments of mitochondrial function [17,18,19,20,35].

### 4.3. Respirometry

The principle of respirometry is based on mitochondrial oxygen consumption. Within a closed reaction chamber, oxygen concentrations are recorded continuously. The decline of O_2_-concentration over time allows for assessment of mitochondrial activity in the chamber, and mitochondrial function can be determined using different substrates, uncouplers, and inhibitors of the respiratory system [36]. The oxygen concentration is measured by a Clark-type electrode, which was introduced in 1962 [37]. It consists of a gold or platinum cathode and a silver anode separated from the reaction solution by a semipermeable membrane, which allows O_2_ to diffuse freely, while large molecules are retained. [36]. High-resolution respirometry (HRR) can be carried out using an Oxygraph-2k (Oxygraph-2k, Oroboros Instruments, Innsbruck, Austria). In a 2 ml reaction chamber containing the detector electrode and a stirrer, measurements can be performed at a physiological temperature of 37 °C. The activity of the biological sample in the medium is assessed from the decline of the oxygen concentration in the chamber and the corresponding O_2_ flux (pmol per second per amount of sample) is recorded. The system allows for analysis of integrated mitochondrial function of biological samples using specific substrate-uncoupler-inhibitor titration protocols [38]. In order to reconstitute the citric acid cycle, physiological substrate combinations supporting the NADH, and succinate pathways are added to the chamber. By doing so, non-phosphorylating LEAK respiration (electron flow compensating for proton leak), OXPHOS capacity (maximally ADP-stimulated respiration), and electron transfer (ET) capacity (maximum respiration induced by addition of uncouplers, such as carbonyl cyanide chlorphenylhydrazon (CCCP), carbonyl cyanide-*p*-trifluoromethoxyphenylhydrazone (FCCP) or 2,4-dinitrophenol (DNP)) can be obtained. This approach allows for sensitive analysis of integrated mitochondrial function and provision of diagnostic information on specific mitochondrial impairments in the context of cardiovascular pathologies.

#### Assessment of Reactive Oxygen Species Production

There are many ways to assess ROS production, which have been addressed in previous reviews [39,40]. Generally, assessment of ROS can be achieved via direct and indirect methods. Direct methods, such as electron spin resonance, directly detect free radicals via spin trapping methods, while indirect methods aim at evaluating end products of ROS interactions with lipids, proteins, or nucleic acids as markers of oxidative damage. 

A commonly used way to indirectly assess ROS is via the Amplex Red assay, which detects extracellular H_2_O_2_ by combining N-acetyl-3,7-dihydroxyphenoxazine (Amplex Red) with H_2_O_2_ in a 1:1 stoichiometry to generate a red-fluorescent oxidation product resorufin [41,42]. This assay is very specific and sensitive, but Amplex Red is light sensitive and, hence, susceptible to artefactual resorufin production, if light exposure is not minimized appropriately.

## 5. Surrogate Markers to Evaluate Mitochondrial Content in PAD

Changes of mitochondrial function in response to interventions or pathologies can be rooted in sole alterations of mitochondrial content or may also involve qualitative changes or other factors, such as changes of proteins, fission-fusion events, membrane integrity, cristae formation, or membrane potential [43]. It is, therefore, critical in bioenergetic research to assess mitochondrial content in order to obtain information on intrinsic mitochondrial-specific changes. Transmission electron microscopy (TEM) assesses mitochondrial fractional area, which is an established gold standard marker for mitochondrial content [44,45]. In the light of high costs, and requirements of elaborated instrumentation and time, other surrogate markers of mitochondrial content have been used, which are more easily attainable. These include citrate synthase (CS) activity, cardiolipin content, mitochondrial DNA content, complex I–V protein content, and complex I–IV activity [46]. In a general population of healthy males, cardiolipin content was the best predictor of mitochondrial content assessed from TEM, followed by CS and complex I activity [46]. Although these markers are commonly used in clinical research, their validity and predictive value may vary in different clinical conditions. In a collective of PAD patients with critical limb ischemia, neither CS, nor cytochrome c oxidase subunit IV or 3-hydroxyacyl-CoA dehydrogenase, could predict mitochondrial content assessed from TEM [47]. This study shows that certain markers used in healthy individuals cannot readily be extrapolated to be used in PAD patients.

Although the bioenergetic signature of healthy individuals may differ from that of claudicating PAD patients or patients with critical limb ischemia [47], mitochondrial content was similar across these cohorts [47,48]. Of note, others found higher total mitochondrial volume densities assessed via TEM between the asymptomatic and symptomatic tibialis muscle in patients with unilateral intermittent claudication [49]. In the gastrocnemius muscle of patients with peripheral arterial insufficiency, both CS and mitochondrial volume density were elevated compared to healthy individuals.

In conclusion, validity of mitochondrial markers derived from healthy populations may not be applicable to patients with cardiovascular disease. The effect of PAD on mitochondrial content depends on the disease state of the patient and the sampled muscle, as well as the comparator (asymptomatic leg or control population). Considering these factors, future research will help to derive valid mitochondrial markers for individuals affected by PAD.

## 6. Clinical Relevance of Mitochondrial Recovery in PAD

Effects of revascularization procedures on hemodynamic and functional outcomes of affected lower extremities are well established. Regensteiner et al. demonstrated an improved resting ABI and a relief of intermittent claudication leading to better exercise performance, as well as self-reported community-based walking ability, after surgical revascularization procedures in patients with mild forms of PAD [8]. In contrast, in patients with critical limb ischemia who received infrainguinal revascularization, there was no benefit in functional status, despite the success of limb salvage [7], leading to the assumption of irreversible changes of the muscle structure in the chronic disease setting. A recently published meta-analysis evaluated the effect of different treatment strategies (cliostazol, home-based exercise therapy, supervised exercise therapy, endovascular revascularization, and a combination of treatment options) on short-, mid-, and long-term outcomes. The authors reported improved maximal walking distance in patients receiving either supervised exercise training, endovascular revascularization, or a combination of these two modalities up to the two-year follow-up but not beyond this time point [49]. Further research about the effect of revascularization procedures showed no or only a mild effect on muscle metabolism assessed from PCr recovery rates by ^31^P-MRS [16,50]. Missing information on time interval or utilization of a relatively short time intervals between revascularization procedures and subsequent ^31^P-MRS measurements question the value of these findings for assessment of bioenergetics changes in the context of revascularization procedures. The observation of mitochondrial plasticity in response to medical treatment was supported by data from Pipinos et al. [51]. Non-diabetic patients presenting intermittent claudication were treated with the xanthine derivate pentoxyfylline. After an interval of 12 weeks, ^31^P-MRS measurements demonstrated a direct correlation between functional outcomes (treadmill test) and PCr recovery rates [51]. Therefore, further investigation of therapeutic and pharmacological measures impacting hemodynamics and mitochondrial function is of great interest in light of these promising findings.

When assessing mitochondrial recovery after successful treatment of underlying arteriosclerotic lesions, increased physical activity levels need to be considered as a factor aiding mitochondrial improvements. Patients suffering from symptomatic PAD are characterized by decreased physical activity levels caused by walking-induced pain. The goal of revascularization procedures in these patients is to reestablish blood supply to affected muscle regions in the course of treating flow-limited arteriosclerotic lesions. As a result, intermittent claudication is dissolved, and patients may increase physical activity levels as walking performances improve post-surgery [52]. Mitochondria are able to improve their content, as well as respiratory capacity, following increased metabolic demand from exercise training [53]. Different exercise training modalities result in improved mitochondrial function assessed by high-resolution respirometry [54]. While restricted physical activity will result in deconditioned mitochondria, increasing physical activity levels after dissolving intermittent claudication will likely impact mitochondrial respiration after successful treatment. Besides the treatment option of revascularization procedures, exercise training can contribute to development of arterial collateral networks and improve distal perfusion and limit walking-induced pain. Exercise is therefore recommended in patients with mild forms of PAD [6]. Several authors demonstrated the beneficial effects of supervised exercise training on quality of life of patients as walking performances were improved [55,56]. Data about the isolated effect of exercise training on mitochondrial function in PAD is scarce so far. A Norwegian group investigated the effect of a defined exercise training program over 8 weeks on mitochondrial function and clinical outcomes in patients suffering from intermittent claudication. Depending on the effect on maximal walking distances, patients were divided into responders or non-responders. The group of non-responders also did not improve their respiratory function [57]. We assessed the effect of revascularization procedures on mitochondrial function in patients with mild forms of PAD and the impact on quality of life. We were able to demonstrate that in a group of patients suffering from mild forms of PAD (Fontaine stage IIB and III), successful revascularization procedures (open surgery and endovascular procedures) lead to a recovery of initially impaired mitochondrial function after 6 weeks [58]. Interestingly, our results demonstrated that initially reduced mitochondrial content improved after revascularization. Six weeks after successful revascularization, parameters of mitochondrial function (mitochondrial content and respiration) improved in the patients to a level comparable to healthy control individuals. This led us to the conclusion that mitochondrial plasticity allows for mitochondrial recovery, at least in mild forms of PAD [58]. As we solely included patients with flow-limiting pathologies of the superficial artery, we defined ischemic (gastrocnemius muscle) and non-ischemic (vastus lateralis muscle) muscle regions [59]. Differences were restricted to ischemic muscle regions, while non-ischemic muscle regions did not show any changes in the measured parameters. In this cohort, we were able to demonstrate that the effect of the restoration of blood supply has a greater impact on mitochondrial recovery than an increase of physical activity levels [59]. The exact mechanisms and the contributory role of ROS within the recovery of mitochondria after revascularization procedures need to be investigated in upcoming trials. With the focus on patients suffering from lifestyle limiting intermittent claudication, it will be important to identify associations of mitochondrial markers with clinical outcomes. Table 1 provides an overview of clinical studies on mitochondrial function in PAD.

## 7. Summary and Conclusions

The evidence of mitochondrial alterations in patients suffering from PAD is based on a number of clinical studies using a range of in-vivo and in-vitro methods (Table 1). However, mitochondria are able to recover after revascularization procedures, and the identification of mitochondrial pathways being relevant in this process is an important topic of future trials with special focus on the role of ROS in mitochondrial recovery. Both revascularization procedures, as well as exercise training, remain cornerstone therapies for improving PAD, with pharmacological options gaining ground. The correlation of mitochondrial alterations with clinical outcomes enables the declaration of patients who might benefit from early revascularization procedures by identification of specific mitochondrial lesions.

## Figures and Tables

**Table 1:** Overview of clinical studies investigating mitochondrial function in PAD.

Reference	Methods	PAD Severity	Outcome/Conclusion
Zatina et al. 1986 [19]	^31^P MRS before and after revascularization;	Not specified;	^31^P MRS successfully measures impaired bioenergetics of ischemic limbs during exercise and recovery;
Pipinos et al. 2000 [20]	^31^P MRS before and after isometric exercise;	Stage IIB PAD;	↑phosphocreatine and ADP recovery time constants in claudicating calf muscle;
Pipinos et al. 2002 [51]	^31^P MRS before and after 12 weeks of interval exercise training;	Stage IIB PAD;	Pentoxifilline improves mitochondriopathy of claudicating muscle;
Pipinos et al. 2002 [21]	In-vitro respirometry;	Advanced PAD;	↓mitochondrial respiration in patients suffering from advanced PAD;
Greiner et al. 2006 [18]	Serial ^31^P MRS during incremental exercise;	Symptomatic unilateral PAD;	↑PCr recovery time after unilateral exercise in claudicating calf muscle;
Pipinos et al. 2006 [22]	Enzymatic activity measurement, in-vitro respirometry;	Severe PAD;	↓mitochondrial respiration and enzymatic activities of complexes I, III, and IV in PAD; ↑oxidative stress biomarkers, ↓antioxidative enzymes;
Van Schaardenburgh et al. [57]	In-vitro respirometry;	Stage IIB PAD;	Changes in walking performances relate to changes in mitochondrial function after exercise;
Gratl et al. 2020 [58]	In-vitro respirometry;	Stage IIB/III PAD	Mitochondrial recovery after successful revascularization;
Gratl et al. 2021 [59]	In-vitro respirometry;	Stage IIB/III PAD	Restoration of blood supply is more important to mitochondrial recovery than increased physical activity
